# Neoaustin: a meroterpene produced by *Penicillium sp*.

**DOI:** 10.1107/S1600536809006618

**Published:** 2009-02-25

**Authors:** Julio Zukerman-Schpector, Stella H. Maganhi, Taicia Pacheco Fill, Edson Rodrigues-Fo, Ignez Caracelli

**Affiliations:** a Department of Chemistry, Universidade Federal de São Carlos, 13565-905 São Carlos, SP, Brazil; b Physics Department, Universidade Estadual Paulista, "Júlio de Mesquita Filho", UNESP, 17033-360 Bauru, SP, Brazil

## Abstract

The title meroterpene neoaustin {systematic name: (1′*S*,2′*R*,3*S*,7′*R*,9′*S*,11′*S*,12′*R*)-11′-hydr­oxy-2,2,2′,9′,12′-penta­methyl-6′,15′-dimethyl­ene-2,6-dihydro-13′-oxaspiro­[pyran-3,5′-tetra­cyclo­[7.5.1.0^1,11^.0^2,7^]penta­deca­ne]-6,10′,14′-trione}, C_25_H_30_O_6_, comprises five rings, three six-membered and two five-membered. The absolute configuration was established based on [α_D_] = +166.91° (*c* 1.21, CH_2_Cl_2_). In the crystal, the mol­ecules are connected into a supra­molecular helical chain *via* O—H⋯O hydrogen bonds reinforced by C—H⋯O contacts.

## Related literature

For related literature, see: dos Santos & Rodrigues-Fo (2002 ▶, 2003 ▶); Maganhi *et al.* 2009 ▶. For ring conformation analysis, see: Cremer & Pople (1975 ▶); Iulek & Zukerman-Schpector (1997 ▶).
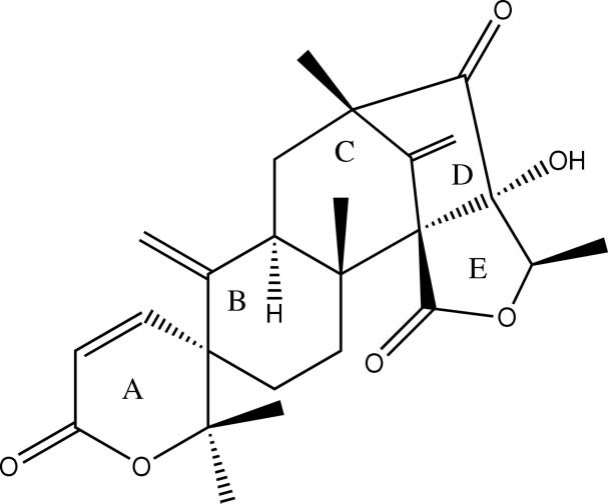


## Experimental

### 

#### Crystal data

C_25_H_30_O_6_
*M* *_r_* = 426.49
Orthorhombic, 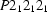

*a* = 11.2152 (4) Å
*b* = 13.2870 (5) Å
*c* = 14.3914 (7) Å
*V* = 2144.55 (15) Å^3^
*Z* = 4
Mo *K*α radiation
μ = 0.09 mm^−1^
*T* = 290 K
0.49 × 0.39 × 0.21 mm

#### Data collection

Bruker APEXII CCD area-detector diffractometer
Absorption correction: none
18157 measured reflections
2622 independent reflections
2453 reflections with *I* > 2σ(*I*)
*R*_int_ = 0.048

#### Refinement

*R*[*F* ^2^ > 2σ(*F* ^2^)] = 0.039
*wR*(*F* ^2^) = 0.106
*S* = 1.07
2622 reflections
286 parameters
H-atom parameters constrained
Δρ_max_ = 0.17 e Å^−3^
Δρ_min_ = −0.13 e Å^−3^

### 

Data collection: *APEX2*, *COSMO* and *BIS* (Bruker, 2006 ▶); cell refinement: *SAINT* (Bruker, 2006 ▶); data reduction: *SAINT*; program(s) used to solve structure: *SIR97* (Altomare *et al.*, 1999 ▶); program(s) used to refine structure: *SHELXL97* (Sheldrick, 2008 ▶); molecular graphics: *ORTEP-3* (Farrugia, 1997 ▶); software used to prepare material for publication: *WinGX* (Farrugia, 1999 ▶) and *PARST* (Nardelli, 1995 ▶).

## Supplementary Material

Crystal structure: contains datablocks global, I. DOI: 10.1107/S1600536809006618/tk2374sup1.cif

Structure factors: contains datablocks I. DOI: 10.1107/S1600536809006618/tk2374Isup2.hkl

Additional supplementary materials:  crystallographic information; 3D view; checkCIF report

## Figures and Tables

**Table 1:** Hydrogen-bond geometry (Å, °)

*D*—H⋯*A*	*D*—H	H⋯*A*	*D*⋯*A*	*D*—H⋯*A*
O4—H1*O*4⋯O2^i^	0.82	2.06	2.852 (3)	162
C5—H5⋯O3^ii^	0.93	2.63	3.386 (3)	139

Symmetry codes: (i) 
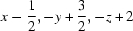
; (ii) 
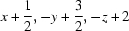
.

**Fig. 1.**
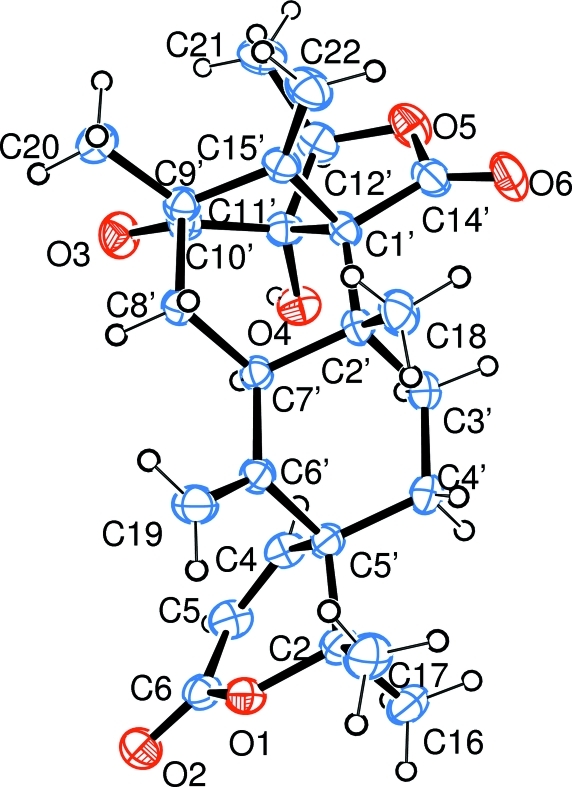
The molecular structure of (I) showing atom labelling scheme and displacement ellipsoids at the 30% probability level (arbitrary spheres for the H atoms).

**Fig. 2.**
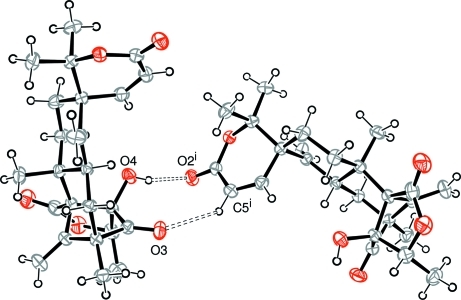
Detail of the hydrogen bonding in (I). Hydrogen bonds are shown as hollow dashed bonds. See Table 1 for symmetry operations.

## supplementary crystallographic information

### Comment

Endophytic fungi live in very intimate association with plant tissue and can produce compounds similar and sometimes identical to those produced by the host plant. Thus, fungi have been a rich source of important biologically active secondary metabolites, such as meroterpenoids, a class of complex metabolites derived from a mixed terpenoid-polyketide biosynthetic pathway. During an on-going study of substances produced by endophytic fungi, the title compound (I) was isolated and its structure postulated based on APCIMS (Atmospheric Pressure Chemical Ionization Mass Spectrometry) and a variety of NMR studies (dos Santos and Rodrigues-Fo, 2003). As suitable crystals were subsequently obtained, a crystal structure determination of (I) was undertaken, Fig. 1. The three six-membered rings are in different distorted conformations. Referring to the labels in Scheme 1, ring A is in a highly distorted half-boat conformation, ring B in a slightly distorted chair, and ring C is in a chair distorted towards a half-chair conformation. The five membered rings, D and E, are in a highly distorted envelope and a distorted twist conformation, respectively. The ring-puckering parameters (Cremer & Pople, 1975; Iulek & Zukerman-Schpector, 1997) in the order for A, B, C, D and E (when applicable) are: q_2_ = 0.434 (2), 0.044 (2), 0.161 (2), 0.562 (2), 0.284 (2) Å, q_3_ = 0.241 (2), 0.552 (2), -0.650 (2) Å, Q = 0.496 (2), 0.554 (2), 0.669 (2)°, φ_2_ =-73.0 (3), -36 (3), 146.7 (7), -154.3 (3), 25.1 (5)°, and θ_2_ = 60.9 (3), 4.5 (2), 166.1 (2)°. The absolute configuration was established based on the [α_D_] = +166.914.97° (c 1.21, CH_2_Cl_2_) and the results reported in dos Santos and Rodrigues-Fo (2003). The molecules are linked *via* O-H···O hydrogen bonds, Fig. 2. which extend into a supramolecular helical chain which is reinforced via C-H···O contacts (Table 1).

### Experimental

Compound (I), Neoaustin, was produced during cultivation of the fungus *Penicillum sp* over sterilized rice, and isolated from the methanol extract of the culture. Suitable crystals were obtained, by slow evaporation, from a mixture of dichloromethane, methanol and water.

### Refinement

The H atoms were refined in the riding-model approximation with C—H = 0.93 - 0.98 Å and (0.82 Å for O—H), and with *U*_iso_(H) = 1.5*U*_eq_(methyl-C) or 1.2*U*_eq_(remaining-C and O). In the absence of significant anomalous scattering effects, 1008 Friedel pairs were averaged in the final refinement.

### Crystal data

**Table:**

C_25_H_30_O_6_	*F*(000) = 912
*M**_r_* = 426.49	*D*_x_ = 1.321 Mg m^−^^3^
Orthorhombic, *P*2_1_2_1_2_1_	Mo *K*α radiation, λ = 0.71073 Å
Hall symbol: P 2ac 2ab	Cell parameters from 33851 reflections
*a* = 11.2152 (4) Å	θ = 1.0–27.4°
*b* = 13.2870 (5) Å	µ = 0.09 mm^−^^1^
*c* = 14.3914 (7) Å	*T* = 290 K
*V* = 2144.55 (15) Å^3^	Prism, colorless
*Z* = 4	0.49 × 0.39 × 0.21 mm

### Data collection

**Table:**

Bruker APEXII CCD area-detector diffractometer	2453 reflections with *I* > 2σ(*I*)
Radiation source: fine-focus sealed tube	*R*_int_ = 0.048
graphite	θ_max_ = 27.0°, θ_min_ = 3.2°
φ and ω scans	*h* = −14→14
18157 measured reflections	*k* = −15→16
2622 independent reflections	*l* = −17→18

### Refinement

**Table:**

Refinement on *F*^2^	Primary atom site location: structure-invariant direct methods
Least-squares matrix: full	Secondary atom site location: difference Fourier map
*R*[*F*^2^ > 2σ(*F*^2^)] = 0.039	Hydrogen site location: inferred from neighbouring sites
*wR*(*F*^2^) = 0.106	H-atom parameters constrained
*S* = 1.07	*w* = 1/[σ^2^(*F*_o_^2^) + (0.0607*P*)^2^ + 0.3107*P*] where *P* = (*F*_o_^2^ + 2*F*_c_^2^)/3
2622 reflections	(Δ/σ)_max_ < 0.001
286 parameters	Δρ_max_ = 0.17 e Å^−^^3^
0 restraints	Δρ_min_ = −0.13 e Å^−^^3^

### Special details

**Table:**

Geometry. All e.s.d.'s (except the e.s.d. in the dihedral angle between two l.s. planes) are estimated using the full covariance matrix. The cell e.s.d.'s are taken into account individually in the estimation of e.s.d.'s in distances, angles and torsion angles; correlations between e.s.d.'s in cell parameters are only used when they are defined by crystal symmetry. An approximate (isotropic) treatment of cell e.s.d.'s is used for estimating e.s.d.'s involving l.s. planes.
Refinement. Refinement of *F*^2^ against ALL reflections. The weighted *R*-factor *wR* and goodness of fit *S* are based on *F*^2^, conventional *R*-factors *R* are based on *F*, with *F* set to zero for negative *F*^2^. The threshold expression of *F*^2^ > σ(*F*^2^) is used only for calculating *R*-factors(gt) *etc*. and is not relevant to the choice of reflections for refinement. *R*-factors based on *F*^2^ are statistically about twice as large as those based on *F*, and *R*- factors based on ALL data will be even larger.

### Fractional atomic coordinates and isotropic or equivalent isotropic displacement parameters (Å^2^)

**Table:**

	*x*	*y*	*z*	*U*_iso_*/*U*_eq_	
C1'	0.32891 (19)	0.90837 (15)	0.67021 (13)	0.0371 (4)	
C2'	0.45203 (19)	0.93770 (16)	0.71346 (13)	0.0380 (4)	
C2	0.74745 (19)	0.93484 (17)	0.92112 (15)	0.0425 (5)	
C3'	0.5381 (2)	0.84818 (18)	0.71741 (15)	0.0462 (5)	
H3'A	0.5613	0.8301	0.6546	0.055*	
H3'B	0.4973	0.7909	0.7444	0.055*	
C4'	0.6507 (2)	0.8705 (2)	0.77441 (16)	0.0485 (5)	
H4'A	0.6980	0.9201	0.7415	0.058*	
H4'B	0.6977	0.8094	0.7788	0.058*	
C4	0.5674 (2)	0.82925 (17)	0.93270 (16)	0.0436 (5)	
H4	0.5162	0.7834	0.9046	0.052*	
C5	0.5873 (2)	0.82231 (18)	1.02291 (17)	0.0493 (5)	
H5	0.5532	0.7698	1.0564	0.059*	
C5'	0.62593 (18)	0.90991 (16)	0.87378 (14)	0.0378 (4)	
C6	0.66206 (19)	0.89585 (17)	1.07096 (15)	0.0431 (5)	
C6'	0.53593 (18)	0.99769 (15)	0.86945 (13)	0.0361 (4)	
C7'	0.42380 (18)	0.97177 (15)	0.81504 (13)	0.0346 (4)	
H7'	0.3896	0.9127	0.8459	0.041*	
C8'	0.32623 (19)	1.05317 (15)	0.81811 (14)	0.0375 (4)	
H8'A	0.3586	1.1156	0.7940	0.045*	
H8'B	0.3035	1.0647	0.8823	0.045*	
C9'	0.21293 (19)	1.02470 (16)	0.76119 (14)	0.0385 (4)	
C10'	0.18022 (19)	0.91858 (16)	0.79257 (14)	0.0405 (4)	
C11'	0.24352 (19)	0.84332 (16)	0.72965 (14)	0.0402 (5)	
C12'	0.1604 (2)	0.79507 (18)	0.65534 (17)	0.0523 (6)	
H12'	0.1481	0.7246	0.6731	0.063*	
C14'	0.3301 (2)	0.85332 (18)	0.57786 (15)	0.0493 (5)	
C15'	0.2536 (2)	1.00412 (16)	0.66311 (14)	0.0385 (4)	
C16	0.8344 (2)	0.8465 (2)	0.9222 (2)	0.0571 (6)	
H16A	0.7958	0.7885	0.9482	0.086*	
H16B	0.9027	0.8636	0.9593	0.086*	
H16C	0.8596	0.8319	0.8599	0.086*	
C17	0.8129 (2)	1.0257 (2)	0.8822 (2)	0.0573 (6)	
H17A	0.8738	1.0463	0.9250	0.086*	
H17B	0.7575	1.0799	0.8732	0.086*	
H17C	0.8487	1.0083	0.8238	0.086*	
C18	0.5105 (2)	1.0233 (2)	0.65818 (16)	0.0507 (5)	
H18A	0.5192	1.0033	0.5944	0.076*	
H18B	0.5875	1.0379	0.6840	0.076*	
H18C	0.4613	1.0823	0.6615	0.076*	
C19	0.5471 (2)	1.08493 (18)	0.91461 (17)	0.0516 (5)	
H19A	0.4854	1.1317	0.9133	0.062*	
H19B	0.6166	1.0990	0.9474	0.062*	
C20	0.1151 (2)	1.10233 (19)	0.77492 (17)	0.0498 (5)	
H20A	0.0473	1.0847	0.7375	0.075*	
H20B	0.1439	1.1674	0.7567	0.075*	
H20C	0.0922	1.1039	0.8392	0.075*	
C21	0.0390 (3)	0.8406 (2)	0.6378 (2)	0.0641 (7)	
H21A	−0.0015	0.8026	0.5906	0.096*	
H21B	0.0481	0.9090	0.6176	0.096*	
H21C	−0.0069	0.8391	0.6941	0.096*	
C22	0.2290 (3)	1.05608 (19)	0.58737 (17)	0.0557 (6)	
H22A	0.1811	1.1131	0.5910	0.067*	
H22B	0.2596	1.0357	0.5303	0.067*	
O1	0.72595 (13)	0.95988 (11)	1.02022 (10)	0.0418 (3)	
O2	0.66745 (17)	0.90141 (15)	1.15505 (11)	0.0577 (4)	
O3	0.11959 (17)	0.89618 (14)	0.85870 (12)	0.0583 (5)	
O4	0.30164 (16)	0.76719 (12)	0.78090 (12)	0.0516 (4)	
H1O4	0.2520	0.7281	0.8019	0.062*	
O5	0.23077 (19)	0.79543 (15)	0.57041 (12)	0.0613 (5)	
O6	0.4016 (2)	0.85761 (16)	0.51643 (11)	0.0659 (5)	

### Atomic displacement parameters (Å^2^)

**Table:**

	*U*^11^	*U*^22^	*U*^33^	*U*^12^	*U*^13^	*U*^23^
C1'	0.0436 (10)	0.0378 (9)	0.0299 (8)	−0.0005 (9)	0.0004 (9)	0.0024 (7)
C2'	0.0402 (10)	0.0421 (10)	0.0317 (9)	0.0003 (9)	0.0038 (8)	−0.0007 (8)
C2	0.0336 (10)	0.0503 (12)	0.0435 (11)	0.0003 (9)	0.0020 (9)	0.0028 (9)
C3'	0.0464 (12)	0.0499 (12)	0.0422 (11)	0.0090 (10)	0.0022 (10)	−0.0110 (9)
C4'	0.0429 (11)	0.0544 (12)	0.0482 (12)	0.0104 (10)	0.0038 (10)	−0.0104 (10)
C4	0.0383 (10)	0.0390 (10)	0.0535 (12)	−0.0006 (9)	−0.0035 (10)	0.0038 (9)
C5	0.0471 (12)	0.0477 (12)	0.0531 (12)	−0.0053 (10)	−0.0009 (11)	0.0148 (10)
C5'	0.0334 (9)	0.0408 (10)	0.0393 (10)	0.0018 (8)	0.0004 (8)	0.0023 (8)
C6	0.0386 (10)	0.0461 (11)	0.0445 (11)	0.0052 (9)	−0.0007 (9)	0.0082 (9)
C6'	0.0365 (9)	0.0385 (10)	0.0332 (9)	0.0030 (8)	0.0015 (8)	0.0028 (7)
C7'	0.0360 (9)	0.0357 (9)	0.0320 (9)	0.0027 (8)	0.0024 (7)	0.0013 (7)
C8'	0.0384 (10)	0.0386 (10)	0.0356 (9)	0.0036 (9)	0.0000 (8)	−0.0013 (7)
C9'	0.0373 (10)	0.0412 (10)	0.0369 (9)	0.0012 (8)	−0.0006 (8)	0.0029 (8)
C10'	0.0387 (10)	0.0460 (11)	0.0368 (10)	−0.0025 (9)	−0.0023 (9)	0.0045 (9)
C11'	0.0440 (11)	0.0361 (10)	0.0405 (10)	−0.0009 (9)	−0.0015 (9)	0.0073 (8)
C12'	0.0624 (14)	0.0418 (11)	0.0526 (12)	−0.0092 (11)	−0.0059 (12)	−0.0023 (10)
C14'	0.0629 (14)	0.0479 (12)	0.0372 (10)	0.0058 (11)	−0.0052 (11)	−0.0036 (9)
C15'	0.0431 (10)	0.0366 (10)	0.0358 (9)	−0.0009 (8)	−0.0033 (9)	0.0025 (8)
C16	0.0382 (11)	0.0655 (15)	0.0676 (15)	0.0106 (11)	−0.0022 (12)	−0.0038 (13)
C17	0.0458 (12)	0.0654 (15)	0.0607 (14)	−0.0140 (12)	0.0047 (12)	0.0108 (12)
C18	0.0514 (12)	0.0591 (13)	0.0416 (11)	−0.0083 (11)	0.0096 (11)	0.0053 (10)
C19	0.0544 (13)	0.0462 (12)	0.0543 (12)	0.0071 (11)	−0.0140 (11)	−0.0064 (10)
C20	0.0426 (11)	0.0535 (13)	0.0533 (12)	0.0095 (11)	−0.0022 (11)	0.0008 (10)
C21	0.0595 (15)	0.0670 (16)	0.0657 (16)	−0.0123 (14)	−0.0194 (13)	−0.0019 (13)
C22	0.0767 (17)	0.0485 (12)	0.0418 (11)	0.0053 (12)	−0.0051 (12)	0.0092 (10)
O1	0.0388 (7)	0.0459 (8)	0.0405 (7)	−0.0034 (7)	−0.0043 (6)	0.0034 (6)
O2	0.0596 (10)	0.0723 (11)	0.0413 (8)	0.0051 (10)	0.0001 (8)	0.0080 (8)
O3	0.0566 (10)	0.0651 (11)	0.0531 (9)	−0.0069 (9)	0.0156 (9)	0.0095 (8)
O4	0.0579 (9)	0.0399 (8)	0.0569 (9)	−0.0017 (7)	−0.0022 (8)	0.0156 (7)
O5	0.0738 (12)	0.0619 (10)	0.0482 (9)	−0.0079 (10)	−0.0056 (9)	−0.0151 (8)
O6	0.0809 (13)	0.0804 (13)	0.0363 (8)	0.0021 (11)	0.0085 (9)	−0.0096 (8)

### Geometric parameters (Å, °)

**Table:**

C1'—C14'	1.517 (3)	C9'—C20	1.519 (3)
C1'—C15'	1.530 (3)	C9'—C10'	1.525 (3)
C1'—C11'	1.548 (3)	C10'—O3	1.207 (3)
C1'—C2'	1.564 (3)	C10'—C11'	1.524 (3)
C2'—C3'	1.533 (3)	C11'—O4	1.411 (3)
C2'—C18	1.535 (3)	C11'—C12'	1.557 (3)
C2'—C7'	1.563 (3)	C12'—O5	1.455 (3)
C2—O1	1.484 (3)	C12'—C21	1.511 (4)
C2—C17	1.520 (3)	C12'—H12'	0.9800
C2—C16	1.526 (3)	C14'—O6	1.195 (3)
C2—C5'	1.559 (3)	C14'—O5	1.358 (3)
C3'—C4'	1.534 (3)	C15'—C22	1.319 (3)
C3'—H3'A	0.9700	C16—H16A	0.9600
C3'—H3'B	0.9700	C16—H16B	0.9600
C4'—C5'	1.548 (3)	C16—H16C	0.9600
C4'—H4'A	0.9700	C17—H17A	0.9600
C4'—H4'B	0.9700	C17—H17B	0.9600
C4—C5	1.321 (3)	C17—H17C	0.9600
C4—C5'	1.516 (3)	C18—H18A	0.9600
C4—H4	0.9300	C18—H18B	0.9600
C5—C6	1.461 (3)	C18—H18C	0.9600
C5—H5	0.9300	C19—H19A	0.9300
C5'—C6'	1.544 (3)	C19—H19B	0.9300
C6—O2	1.214 (3)	C20—H20A	0.9600
C6—O1	1.331 (3)	C20—H20B	0.9600
C6'—C19	1.335 (3)	C20—H20C	0.9600
C6'—C7'	1.521 (3)	C21—H21A	0.9600
C7'—C8'	1.539 (3)	C21—H21B	0.9600
C7'—H7'	0.9800	C21—H21C	0.9600
C8'—C9'	1.558 (3)	C22—H22A	0.9300
C8'—H8'A	0.9700	C22—H22B	0.9300
C8'—H8'B	0.9700	O4—H1O4	0.8200
C9'—C15'	1.508 (3)		
			
C14'—C1'—C15'	110.33 (17)	C15'—C9'—C8'	106.81 (17)
C14'—C1'—C11'	102.75 (18)	C20—C9'—C8'	110.84 (17)
C15'—C1'—C11'	99.19 (16)	C10'—C9'—C8'	105.36 (16)
C14'—C1'—C2'	117.43 (19)	O3—C10'—C11'	124.7 (2)
C15'—C1'—C2'	107.85 (16)	O3—C10'—C9'	126.7 (2)
C11'—C1'—C2'	117.74 (16)	C11'—C10'—C9'	108.57 (16)
C3'—C2'—C18	108.96 (18)	O4—C11'—C10'	112.01 (17)
C3'—C2'—C7'	108.52 (16)	O4—C11'—C1'	113.78 (18)
C18—C2'—C7'	110.90 (17)	C10'—C11'—C1'	104.48 (16)
C3'—C2'—C1'	112.18 (17)	O4—C11'—C12'	109.90 (18)
C18—C2'—C1'	110.82 (17)	C10'—C11'—C12'	113.54 (18)
C7'—C2'—C1'	105.40 (16)	C1'—C11'—C12'	102.76 (17)
O1—C2—C17	104.74 (19)	O5—C12'—C21	110.3 (2)
O1—C2—C16	105.43 (18)	O5—C12'—C11'	104.53 (18)
C17—C2—C16	107.8 (2)	C21—C12'—C11'	119.3 (2)
O1—C2—C5'	108.99 (16)	O5—C12'—H12'	107.4
C17—C2—C5'	115.45 (19)	C21—C12'—H12'	107.4
C16—C2—C5'	113.58 (19)	C11'—C12'—H12'	107.4
C2'—C3'—C4'	112.83 (18)	O6—C14'—O5	121.3 (2)
C2'—C3'—H3'A	109.0	O6—C14'—C1'	129.2 (2)
C4'—C3'—H3'A	109.0	O5—C14'—C1'	109.5 (2)
C2'—C3'—H3'B	109.0	C22—C15'—C9'	127.9 (2)
C4'—C3'—H3'B	109.0	C22—C15'—C1'	127.3 (2)
H3'A—C3'—H3'B	107.8	C9'—C15'—C1'	104.78 (16)
C3'—C4'—C5'	114.33 (18)	C2—C16—H16A	109.5
C3'—C4'—H4'A	108.7	C2—C16—H16B	109.5
C5'—C4'—H4'A	108.7	H16A—C16—H16B	109.5
C3'—C4'—H4'B	108.7	C2—C16—H16C	109.5
C5'—C4'—H4'B	108.7	H16A—C16—H16C	109.5
H4'A—C4'—H4'B	107.6	H16B—C16—H16C	109.5
C5—C4—C5'	121.8 (2)	C2—C17—H17A	109.5
C5—C4—H4	119.1	C2—C17—H17B	109.5
C5'—C4—H4	119.1	H17A—C17—H17B	109.5
C4—C5—C6	121.0 (2)	C2—C17—H17C	109.5
C4—C5—H5	119.5	H17A—C17—H17C	109.5
C6—C5—H5	119.5	H17B—C17—H17C	109.5
C4—C5'—C6'	105.89 (16)	C2'—C18—H18A	109.5
C4—C5'—C4'	110.80 (19)	C2'—C18—H18B	109.5
C6'—C5'—C4'	109.60 (16)	H18A—C18—H18B	109.5
C4—C5'—C2	106.50 (17)	C2'—C18—H18C	109.5
C6'—C5'—C2	115.38 (18)	H18A—C18—H18C	109.5
C4'—C5'—C2	108.59 (17)	H18B—C18—H18C	109.5
O2—C6—O1	118.8 (2)	C6'—C19—H19A	120.0
O2—C6—C5	122.7 (2)	C6'—C19—H19B	120.0
O1—C6—C5	118.5 (2)	H19A—C19—H19B	120.0
C19—C6'—C7'	121.66 (19)	C9'—C20—H20A	109.5
C19—C6'—C5'	125.1 (2)	C9'—C20—H20B	109.5
C7'—C6'—C5'	112.97 (16)	H20A—C20—H20B	109.5
C6'—C7'—C8'	114.45 (16)	C9'—C20—H20C	109.5
C6'—C7'—C2'	112.31 (16)	H20A—C20—H20C	109.5
C8'—C7'—C2'	111.99 (15)	H20B—C20—H20C	109.5
C6'—C7'—H7'	105.8	C12'—C21—H21A	109.5
C8'—C7'—H7'	105.8	C12'—C21—H21B	109.5
C2'—C7'—H7'	105.8	H21A—C21—H21B	109.5
C7'—C8'—C9'	113.20 (16)	C12'—C21—H21C	109.5
C7'—C8'—H8'A	108.9	H21A—C21—H21C	109.5
C9'—C8'—H8'A	108.9	H21B—C21—H21C	109.5
C7'—C8'—H8'B	108.9	C15'—C22—H22A	120.0
C9'—C8'—H8'B	108.9	C15'—C22—H22B	120.0
H8'A—C8'—H8'B	107.8	H22A—C22—H22B	120.0
C15'—C9'—C20	117.60 (18)	C6—O1—C2	118.14 (18)
C15'—C9'—C10'	100.50 (17)	C11'—O4—H1O4	109.5
C20—C9'—C10'	114.55 (18)	C14'—O5—C12'	112.37 (17)
			
C14'—C1'—C2'—C3'	−50.6 (2)	C20—C9'—C10'—O3	−36.0 (3)
C15'—C1'—C2'—C3'	−175.97 (16)	C8'—C9'—C10'—O3	86.1 (3)
C11'—C1'—C2'—C3'	73.0 (2)	C15'—C9'—C10'—C11'	20.4 (2)
C14'—C1'—C2'—C18	71.4 (2)	C20—C9'—C10'—C11'	147.45 (18)
C15'—C1'—C2'—C18	−53.9 (2)	C8'—C9'—C10'—C11'	−90.46 (18)
C11'—C1'—C2'—C18	−164.97 (19)	O3—C10'—C11'—O4	−44.7 (3)
C14'—C1'—C2'—C7'	−168.55 (17)	C9'—C10'—C11'—O4	131.88 (18)
C15'—C1'—C2'—C7'	66.12 (19)	O3—C10'—C11'—C1'	−168.3 (2)
C11'—C1'—C2'—C7'	−44.9 (2)	C9'—C10'—C11'—C1'	8.3 (2)
C18—C2'—C3'—C4'	66.8 (2)	O3—C10'—C11'—C12'	80.5 (3)
C7'—C2'—C3'—C4'	−54.1 (2)	C9'—C10'—C11'—C12'	−102.9 (2)
C1'—C2'—C3'—C4'	−170.11 (17)	C14'—C1'—C11'—O4	91.2 (2)
C2'—C3'—C4'—C5'	53.4 (3)	C15'—C1'—C11'—O4	−155.36 (17)
C5'—C4—C5—C6	3.3 (4)	C2'—C1'—C11'—O4	−39.5 (3)
C5—C4—C5'—C6'	−94.1 (3)	C14'—C1'—C11'—C10'	−146.31 (17)
C5—C4—C5'—C4'	147.1 (2)	C15'—C1'—C11'—C10'	−32.90 (19)
C5—C4—C5'—C2	29.2 (3)	C2'—C1'—C11'—C10'	83.0 (2)
C3'—C4'—C5'—C4	66.5 (2)	C14'—C1'—C11'—C12'	−27.5 (2)
C3'—C4'—C5'—C6'	−50.0 (3)	C15'—C1'—C11'—C12'	85.88 (19)
C3'—C4'—C5'—C2	−176.87 (19)	C2'—C1'—C11'—C12'	−158.27 (17)
O1—C2—C5'—C4	−53.3 (2)	O4—C11'—C12'—O5	−96.7 (2)
C17—C2—C5'—C4	−170.8 (2)	C10'—C11'—C12'—O5	137.02 (18)
C16—C2—C5'—C4	63.9 (2)	C1'—C11'—C12'—O5	24.8 (2)
O1—C2—C5'—C6'	63.9 (2)	O4—C11'—C12'—C21	139.5 (2)
C17—C2—C5'—C6'	−53.6 (3)	C10'—C11'—C12'—C21	13.2 (3)
C16—C2—C5'—C6'	−178.92 (18)	C1'—C11'—C12'—C21	−99.0 (2)
O1—C2—C5'—C4'	−172.67 (17)	C15'—C1'—C14'—O6	94.9 (3)
C17—C2—C5'—C4'	69.8 (3)	C11'—C1'—C14'—O6	−160.1 (3)
C16—C2—C5'—C4'	−55.5 (2)	C2'—C1'—C14'—O6	−29.2 (4)
C4—C5—C6—O2	168.2 (2)	C15'—C1'—C14'—O5	−83.3 (2)
C4—C5—C6—O1	−11.2 (3)	C11'—C1'—C14'—O5	21.7 (2)
C4—C5'—C6'—C19	106.6 (2)	C2'—C1'—C14'—O5	152.63 (18)
C4'—C5'—C6'—C19	−133.8 (2)	C20—C9'—C15'—C22	11.9 (4)
C2—C5'—C6'—C19	−10.9 (3)	C10'—C9'—C15'—C22	136.9 (3)
C4—C5'—C6'—C7'	−67.6 (2)	C8'—C9'—C15'—C22	−113.4 (3)
C4'—C5'—C6'—C7'	51.9 (2)	C20—C9'—C15'—C1'	−167.42 (19)
C2—C5'—C6'—C7'	174.84 (16)	C10'—C9'—C15'—C1'	−42.4 (2)
C19—C6'—C7'—C8'	−0.8 (3)	C8'—C9'—C15'—C1'	67.3 (2)
C5'—C6'—C7'—C8'	173.68 (16)	C14'—C1'—C15'—C22	−24.3 (3)
C19—C6'—C7'—C2'	128.4 (2)	C11'—C1'—C15'—C22	−131.7 (3)
C5'—C6'—C7'—C2'	−57.2 (2)	C2'—C1'—C15'—C22	105.1 (3)
C3'—C2'—C7'—C6'	56.4 (2)	C14'—C1'—C15'—C9'	154.94 (19)
C18—C2'—C7'—C6'	−63.2 (2)	C11'—C1'—C15'—C9'	47.58 (19)
C1'—C2'—C7'—C6'	176.80 (16)	C2'—C1'—C15'—C9'	−75.61 (19)
C3'—C2'—C7'—C8'	−173.15 (17)	O2—C6—O1—C2	162.3 (2)
C18—C2'—C7'—C8'	67.2 (2)	C5—C6—O1—C2	−18.3 (3)
C1'—C2'—C7'—C8'	−52.8 (2)	C17—C2—O1—C6	175.49 (19)
C6'—C7'—C8'—C9'	179.61 (16)	C16—C2—O1—C6	−70.9 (2)
C2'—C7'—C8'—C9'	50.3 (2)	C5'—C2—O1—C6	51.4 (2)
C7'—C8'—C9'—C15'	−56.7 (2)	O6—C14'—O5—C12'	175.7 (2)
C7'—C8'—C9'—C20	174.05 (17)	C1'—C14'—O5—C12'	−6.0 (3)
C7'—C8'—C9'—C10'	49.6 (2)	C21—C12'—O5—C14'	117.0 (2)
C15'—C9'—C10'—O3	−163.1 (2)	C11'—C12'—O5—C14'	−12.4 (3)

### Hydrogen-bond geometry (Å, °)

**Table:**

*D*—H···*A*	*D*—H	H···*A*	*D*···*A*	*D*—H···*A*
O4—H1O4···O2^i^	0.82	2.06	2.852 (3)	162
C5—H5···O3^ii^	0.93	2.63	3.386 (3)	139

Symmetry codes: (i) *x*−1/2, −*y*+3/2, −*z*+2; (ii) *x*+1/2, −*y*+3/2, −*z*+2.
